# Sequence-Specific and Visual Identification of the Influenza Virus NS Gene by Azobenzene-Tethered Bis-Peptide Nucleic Acid

**DOI:** 10.1371/journal.pone.0064017

**Published:** 2013-05-21

**Authors:** Kunihiro Kaihatsu, Shinjiro Sawada, Shota Nakamura, Takaaki Nakaya, Teruo Yasunaga, Nobuo Kato

**Affiliations:** 1 The Institute of Scientific and Industrial Research, Osaka University, Osaka, Japan; 2 Research Institute for Microbial Diseases, Osaka University, Osaka, Japan; 3 Department of Infectious Diseases, Kyoto Prefectural University of Medicine, Kyoto, Japan; German Primate Center, Germany

## Abstract

To rapidly and specifically identify highly virulent influenza virus strains, we prepared an azobenzene-tethered hairpin-type peptide nucleic acid, bisPNA-AZO, which has a complementary sequence against a highly conserved genomic RNA sequence within the ribonucleoprotein complex of the 2009 pandemic influenza A virus, H1N1 subtype. bisPNA-AZO recognizes the conserved virus genome sequence in a sequence-specific manner. Immobilization of bisPNA-AZO on a plate allowed capture of the target virus gene and the generation of a visual colour signal.

## Introduction

The threat of an influenza virus pandemic is increasing worldwide, highlighting the need for a diagnostic test kit that can identify the viral strain and its drug resistance. Current methods for identifying influenza virus strains are mainly based on real-time polymerase chain reaction (RT-PCR) technology [1] or immune-chromatography [2]. RT-PCR technology is highly sensitive and provides genome sequence information, but requires costly instruments. In contrast, immune-chromatography allows rapid visualization of the target virus without the use of instruments, but shows cross reactivity and provides no genomic information such as human pathogenicity or drug resistance. Thus, molecular tools that selectively recognize and visualize virus genomes without the need for instruments would enable rapid on-site identification of virus pathogens and their drug resistance.

Peptide nucleic acids (PNAs) are DNA/RNA analogues in which the phosphate backbone has been replaced by a neutral amide backbone composed of N-(2-aminoethyl)glycine linkages. The advantages of PNAs for molecular recognition are their high binding affinity [3], good mismatch discrimination [4], nuclease and protease resistance [5], and low affinity for proteins [6]. These attributes make PNAs particularly useful for detecting viral mRNA in cells [7]. BisPNA-AEEA is composed of two homopyrimidine PNA strands connected via a linker molecule, 2-aminoethoxy-2-ethoxy acetic acid (AEEA). BisPNA-AEEA can invade duplex DNA and form a stable PNA/DNA/PNA triplex with the complementary homopurine strand via Watson-Crick and Hoogsteen base pairing [8]. Modification of cationic peptides on bisPNA further enhanced the triplex formation efficiency of bis-PNA to not only homopurine, but also purine-pyrimidine mixed sequences within duplex DNA [9].

**Figure 1:**
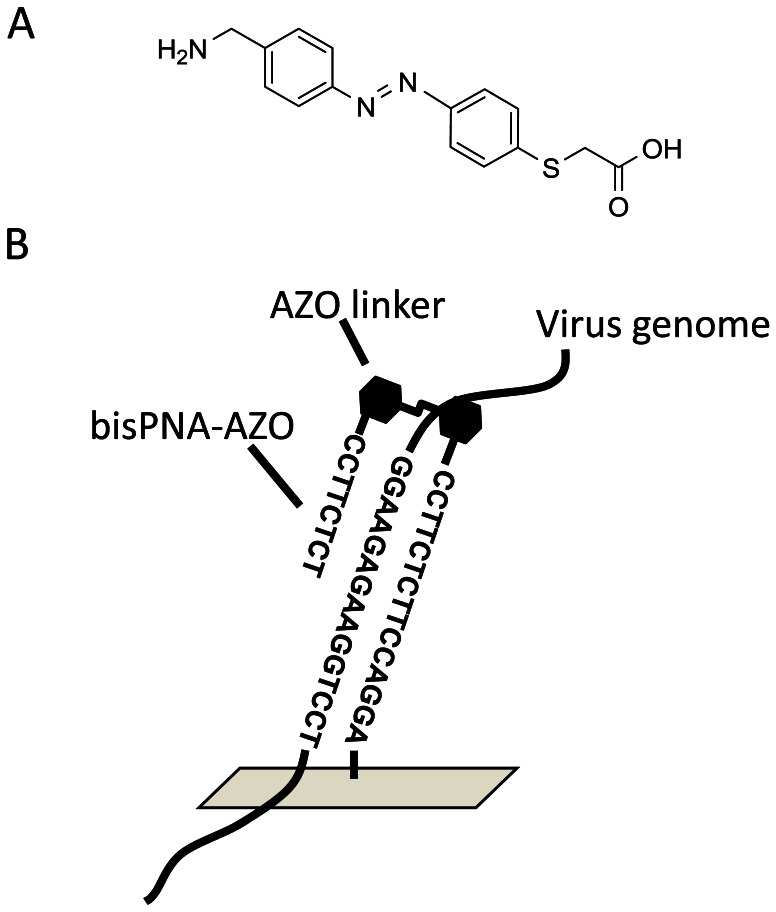
Design of azobenzene-tethered bis-peptide nucleic acid. A) The chemical structure of azobenzene linker (AZO). B) The overall structure of bisPNA-AZO.

In this study, we prepared a newly synthesized bisPNA-AZO which contains an azobenzene amino acid linker [10] instead of the AEEA linker of bisPNA-AEEA (Table 1, Fig. 1). Liang et al. reported that an oligonucleotide tethered to a trans-azobenzene (t-AZO) intercalates between base pairs and stabilizes the duplex by stacking interactions [11]. We expected that introducing azobenzene between two strands of bisPNA would further improve the binding properties of PNA to the target gene. We synthesized a bisPNA-AZO with a sequence complementary to a highly conserved nonstructural protein (NS) gene sequence in swine-origin influenza A/Osaka/180/2009 (H1N1pdm) virus and studied its binding properties to the NS gene.

**Table 1:** Antigene PNA sequences targeting highly conserved sequences in the NS gene of swine-origin influenza A/H1N1 and the HA gene of human pathogenic influenza A/H3N2.

	PNA sequence (N to C)	Mass	
		Calcd.	Found
	Anti-H1N1(NS) PNA		
PNA **1**	H-(Lys)_3_-AEEA-CCTTCTCTTCCAGGA-Lys-(AEEA)_2_-Lys-NH_2_	4682.14	4682.18
PNA **2**	H-(Lys)_3_-TCTCTTCC-AEEA-CCTTCTCTTCCAGGA-Lys-(AEEA)_2_-Lys-NH_2_	7136.88	7137.13
PNA **3**	H-(Lys)_3_-TCTCTTCC-AZO-CCTTCTCTTCCAGGA-Lys-(AEEA)_2_-Lys-NH_2_	7275.09	7299.75
PNA **4**	H-(Lys)_3_-TCTATTCC-AZO-CCTTATCTTCAAGGA-Lys-(AEEA)_2_-Lys-NH_2_	7347.15	7378.55
	Anti-H3N2(HA) PNA		
PNA **5**	H-(Lys)_3_-TTCCCTCC-AEEA-CCTCCCTTAGGTCAC-Lys-(AEEA)_2_-Lys-NH_2_	7106.98	7106.27
PNA **6**	H-(Lys)_3_- TTCCCTCC-AZO-CCTCCCTTAGGTCAC-Lys-(AEEA)_2_-Lys-NH_2_	7245.19	7246.93

AEEA: 2-aminoethoxy-2-ethoxy acetic acid, AZO: thioazobenzene, Lys: lysine.

## Materials and Methods

### Inhibition of Reverse Transcriptase Activity by PNA 1–6

Synthesis of the first strand cDNA for the NS gene of influenza A/Osaka/180/2009 (H1N1pdm) virus required priming with DNA oligonucleotide primer 1∶5′-GGCGTCATGGAAAAGAACAT-3′ (nt 364–383) or primer 2∶5′-CATGACCCTCGAGGAAATGT-3′ (nt 276–295). First, 90 µl of the influenza A virus (6.6 × 10^3^ pfu/ml) and 10 µl of lysis buffer solution were incubated on ice for 30 min. Prior to the extension reaction, 27 µl of the virus solution and 3 µl of PNAs (0, 0.5, 1.0, 2.0 and 4.0 µM) were annealed at 25°C for 30 min, then 1 µl of virus-PNA solution was mixed with a solution containing RT buffer, 5 mM MgCl_2_, 0.5 U RNase inhibitor, 1 mM dNTP and 1 µl of Moloney murine leukemia virus (MuLV) reverse transcriptase (ReverTra Ace, Toyobo, Osaka, Japan) in a final volume of 20 µl. Reverse transcription was performed at 30°C for 10 min, 42°C for 20 min, and stopped by inactivation of the enzymes at 95°C for 5 min.

### Amplification of cDNA of Influenza A Virus Genes by PCR

PCR amplification of influenza A virus genes was carried out using the above-mentioned cDNA samples as templates employing the NS specific primer sets (primer 1 and primer 3∶5′-ACCCCAACTGCATTTTTGAC-3′ (nt 519–539) or primer 2 and primer 4∶5′-ATTGCTCCCTCCTCAGTGAA-3′ (nt 447–466)). This resulted in the amplification of 175 bp (nt 364–539) in the NS gene and 191 bp (nt 276–467) in the HA gene. Amplification of each DNA was performed with a MJ Mini™ thermal cycler (Bio-Rad, Tokyo, Japan) in a volume of 50 µl in 8-well plates. The reaction contained 1 µl of cDNA sample from the above-described reverse transcription, 120 mM Tris–HCl (pH 8.3), 10 mM KCl, 6 mM (NH_4_)_2_SO_4_, 0.1% Triton X-100, 1.5 mM MgCl_2_, 0.2 mM dNTP, 0.2 mM forward and reverse primers, and 1U of KOD-Plus (Toyobo, Osaka, Japan) in a final volume of 50 µl. After 2 min incubation at 94°C, 40 cycles with a temperature profile of 10 s at 98°C, 30 s at 50°C, and 2 min at 68°C were performed in a thermal cycler. DNA amplified at each reaction condition was dissolved in loading buffer containing 30% glycerol, 0.025% bromophenol blue and 0.025% xylencyanol. The mixture was analyzed on a 2.0% agarose gel by electrophoresis. The gel was stained with ethidium bromide and the gel image was observed under UV irradiation.

### Fluorescence Detection of Influenza A Virus on PNA Immobilized Plates

In each well of the ELISA plate, 0.5 µg of PNA was immobilized via primary amine crosslinking. Prior to detection of the influenza A virus genome, the well was pre-warmed with 0.05% Triton-X100-PBS solution at 50°C for 15 min to prevent inter- and intra-molecular aggregation of PNA. At the same time, 90 µl of influenza A or B virus containing 6.6 × 10^2^, 6.6 × 10^3^, 6.6 × 10^4^ or 6.6 × 10^5 ^pfu/ml were mixed with 10 µl of 10% lysis buffer and incubated on ice for 30 min. After removing the Triton-PBS solution from the wells, 100 µl of lysis buffer-treated virus were added to each well and incubated for 1 h at r.t. The wells were washed with 0.05% Triton-X100-PBS (100 µl × 3) and loaded with 20 µl of the manufacturer’s recommended dilution of the anti-influenza A virus nucleoprotein and 20 µl of goat anti-mouse IgG secondary antibody conjugated with HPR in 2% BSA-PBS at a final dilution of 1/20. The wells were placed on an orbital shaker at r.t. for 2 h. After washing the wells with 0.05% Triton-X100-PBS (300 µl × 3), the wells were incubated with an ECL assay solution for 10 or 60 min. HPR conjugated with the anti-mouse IgG secondary antibody produces a stable fluorescent intermediate from TMB in ECL assay solution. This intermediate has an excitation maximum of 430 nm and an emission maximum of 503 nm. This fluorescence spectrum was recorded using a Shimadzu RF-S300PC (Kyoto, Japan) and fluorescence quartz cuvettes (8.5 × 10 mm).

### Visual Detection of Influenza A virus on a PNA Immobilized Plate

In each well of the ELISA plate, 0.5 µg of PNA was immobilized via primary amine crosslinking. Prior to detecting the influenza A virus genome, the well was pre-warmed with 0.05% Triton-X100-PBS solution at 50°C for 15 min to prevent inter- and intra-molecular aggregation of PNA. At the same time, 90 µl of influenza virus containing 6.6 × 10^2^, 6.6 × 10^3^, 6.6 × 10^4^ or 6.6 × 10^5^ pfu/ml were mixed with 10 µl of 10% lysis buffer and incubated on ice for 30 min. After removing the Triton-PBS solution from the wells, 100 µl of the lysis buffer-treated virus were added to each well and incubated for 1 h at r.t. Then, the wells were washed with 0.05% Triton-X100-PBS (100 µl × 3) and were loaded with 20 µl of the manufacturer’s recommended dilution of the anti-influenza A virus nucleoprotein and 20 µl of goat anti-mouse IgG secondary antibody conjugated with alkaline phosphatase in 2% BSA-PBS at a final dilution of 1/20. The wells were placed on an orbital shaker at r.t. for 2 h. After washing the wells with 0.05% Triton-X100-PBS (300 µl × 3), the wells were incubated with a BICP/NBT assay solution for 1 h. Alkaline phosphatase conjugated with the anti-mouse IgG secondary antibody produces a stable purple dye. Images of the plates were taken using a digital camera.

### Absorption vs. Temperature Profiles for bisPNAs Hybridized to ssDNA

Melting profiles of bisPNA (PNA **2–4**) complexed with ssDNA containing the conserved sequence of the NS gene of influenza A/Osaka/180/2009(H1N1pdm) were analysed on a UV1700 spectrophotometer (Shimadzu) using a microcell (8 cell×1 mm) at 260 nm. BisPNA and ssDNA were suspended in Na_2_HPO_4_ buffer (10 mM, pH 6.9) at 5 µM each. The temperature was ramped down from 95 to 10°C at a rate of −1°C/min.

## Results and Discussion

### Design of PNAs with a Sequence Complementary to a Highly Conserved NS Gene Sequence of H1N1pdm

Influenza A virus is a member of the Orthomyxoviridae, a family of enveloped viruses with segmented, single-stranded, negative-sense RNA genomes. The genome of influenza A virus consists of eight single-stranded, negative-sense RNA segments (between 890 and 2341 bases long) that form ribonucleoprotein (RNP) complexes together with a viral RNA (vRNA)-dependent RNA polymerase complex and many nucleoprotein (NP) molecules [12]. The complete genome data of human influenza A viruses were retrieved from the NCBI Influenza Virus Resource. We performed multiple sequence alignments of the sequence data using Multiple Alignment using Fast Fourier Transform (MAFFT) [13] and identified a highly conserved 15-base sequence on the NS gene (99.5%: 3,544 out of 3,561 sequences) of H1N1pdm (nt 490–504, accession number GQ375891). We synthesized PNA **1**, which has only a Watson-Crick strand, and PNA **2–3**, which have both Watson-Crick and Hoogsteen strands, against the conserved sequence in the NS gene (Table 1). PNA **2** contains an AEEA linker while PNA **3** has an azobenzene amino acid linker (Fig. 1). Based on our preliminary study, the AZO linker of PNA **3** contributed to enhanced Hoogsteen basepairing between bisPNA-AZO and its complementary ssDNA (Fig. S1 in File S1). PNA **4** also has the azobenzene amino acid linker between the Watson-Crick and Hoogsteen strands, but its bases and the NS sequence are mismatched. PNA **5** and **6** have both Watson-Crick and Hoogsteen strands against a highly conserved 15-base sequence in the hemagglutinin (HA) gene (97.5%: 3,893 out of 3,993 sequences) for influenza A/H3N2 viruses (nt 365–379, accession number DQ508865) (Table 1). PNA **5** has an AEEA linker between the PNA strands, while PNA **6** contains the azobenzene amino acid instead of AEEA. All PNAs were purified by reverse-phase HPLC (Fig. S2–S7 in File SI) and their molecular weights were confirmed by MALDI-TOF MS analysis (Fig. S8–S13 in File S1).

### PNA Binding Properties for the Influenza A Virus NS Gene

**Figure 2:**
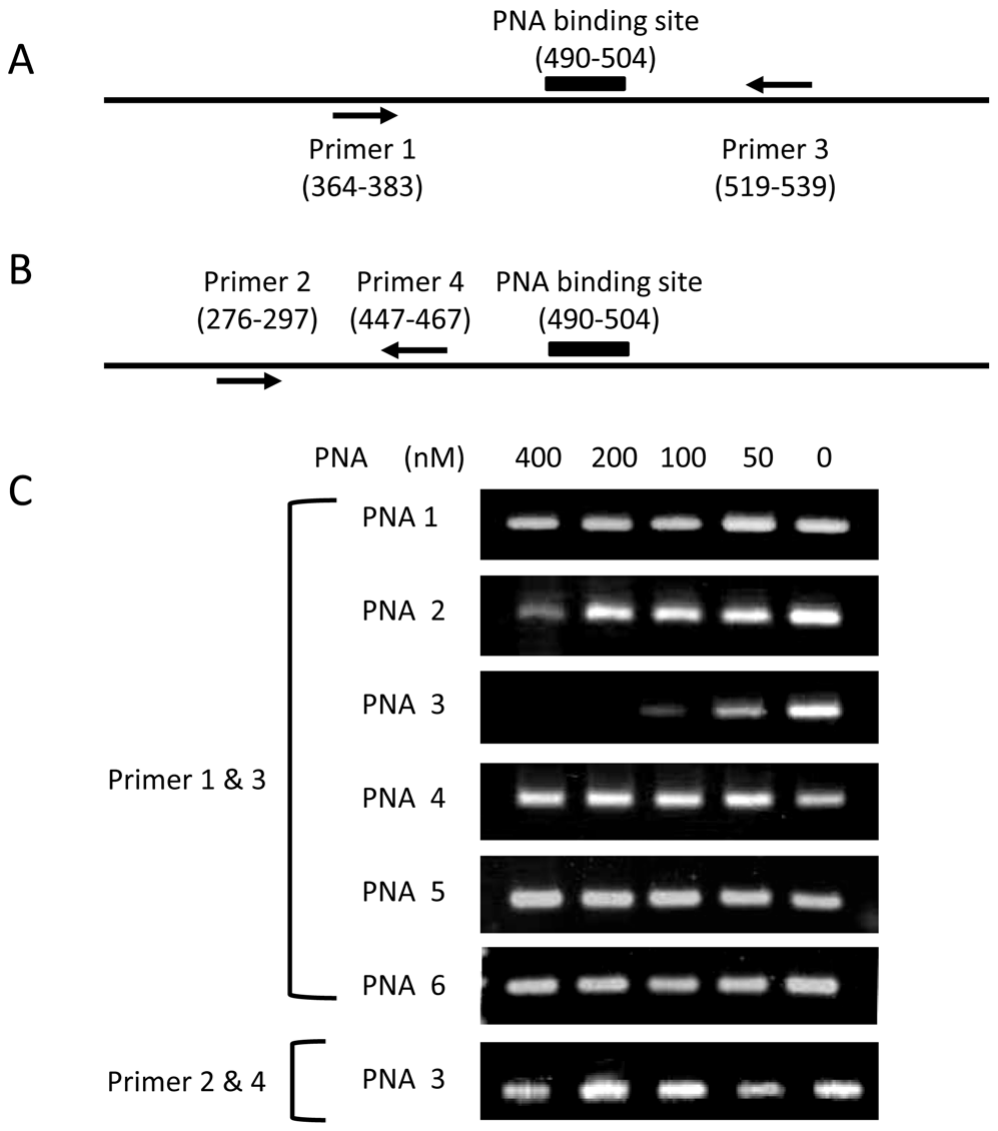
Inhibition of reverse transcription of the NS gene of influenza A/Osaka/180/2009(H1N1) virus by PNA 1–5. A), B) Schematic diagram of the PNA binding site and the primer target sites on the nonstructural protein (NS) gene. Primers 1 and 3 were used to amplify duplex DNA coding nt 364–539 of the NS gene, which contains the PNA binding site. Primers 5 and 6 were used to amplify duplex DNA coding nt 276–297 that does not contain the PNA binding site. PNA 3 inhibited DNA amplification with the primers that amplified duplex DNA containing a PNA binding site on the NS gene. C) Agarose gel analysis of the virus gene transcripts in the presence of the indicated PNA. The NS gene was reverse transcribed into cDNA by Moloney murine leukemia virus (MuLV) reverse transcriptase and then amplified into dsDNA by PCR. The PCR products were analysed by gel electrophoresis on 2% agarose gels. Conditions: 0, 50, 100, 200 or 400 nM of PNAs and 6×10^4^ pfu/ml virus solution were dissolved in 10 mM Na_2_HPO_4_ buffer (pH 6.9) containing 0.1% Triton X-100 solution. Incubation: 25°C for 30 min and then reverse transcribed into cDNA by MuLV reverse transcriptase. The cDNA solution was diluted 50-fold and amplified into dsDNA by a standard PCR reaction.

Influenza A/Osaka/180/2009(H1N1pdm) virus was propagated in chicken embryonated eggs and purified by sucrose gradient ultracentrifugation. For the PNA binding assay, the viruses were incubated with PNA **1–6** in 10 mM Na_2_PO_4_ buffer (pH 6.9) containing 10% lysis solution for 30 min at 25°C. The affinities of PNA **1–6** for the vRNAs were evaluated from their inhibitory effects on cDNA synthesis by MuLV reverse transcriptase. The NS gene of the H1N1pdm virus was reverse-transcribed into cDNA by primer 1 and primer 2, designed for the upper region of the PNA binding site. Thus, cDNA synthesis would terminate if the PNA strongly bound to the vRNA. The resulting cDNA was diluted 50-fold with distilled water and then amplified by polymerase chain reaction (PCR) using either primer 1 and 3 (which contains the PNA binding site) or primer 2 and 4 (which does not contain the PNA binding site) (Fig. 2A,B). The PCR products were analyzed by agarose gel electrophoresis (Fig. 2C). Below 400 nM, PNA **1** did not inhibit reverse transcription of the NS gene of the H1N1pdm virus (Fig. 2C). PNA **2** slightly inhibited reverse transcription at a concentration of 400 nM, whereas PNA **3** strongly inhibited reverse transcription (Fig. 2C). The substitution of the linker moiety of bisPNA from AEEA to the thioazobenzene amino acid improved the inhibitory effect nearly 10-fold. In contrast, PNA **4**, which contains mismatched bases, did not inhibit reverse transcription of the NS gene. Furthermore, PNA **5** and **6**, which are complementary to a conserved sequence of the HA of influenza A/Panama/2007/99(H3N2) virus and contain poly-pyrimidine bases, also did not inhibit reverse transcription of the NS gene of the H1N1pdm virus (Fig. 2C). Thus, PNA **3**, which contains azobenzene as the bisPNA linker, discriminates the target gene sequence and efficiently inhibits the reverse transcription of the NS gene.

### Fluorescence Detection of the Influenza A Virus Genome on a PNA Immobilized Plate

To evaluate whether these PNAs can capture the NS gene, we immobilized 0.5 µg of PNA **1–3** on an enzyme-linked immunosorbent assay (ELISA) plate (Sumitomo Bakelite, Tokyo, Japan). Briefly, primary amine groups of the lysine at the end of the PNA sequences were covalently linked to the surface of a 96-well plate. Each influenza virus solution in 10% lysis buffer was added to the PNA-immobilized plate for 1 h at ambient temperature. Since influenza A virus RNA forms a stable RNP complex with the nucleoprotein (NP) [14], we utilized an ELISA-derived detection system to monitor the NP within the viral RNP complex. The ELISA assay employed a mouse monoclonal anti-NP IgG primary antibody with cross-reactivity to H1–H14 of influenza A/H1N1 viruses, and polyclonal goat anti-mouse IgG secondary antibody linked with horseradish peroxidase (HRP) and its substrate, 3,3′,5,5′-tetramethyl-benzidene (TMB) (Scheme 1). The amount of NS gene captured in each well was monitored by the fluorescence emission at 503 nm of oxidized TMB.

**Figure 3:**
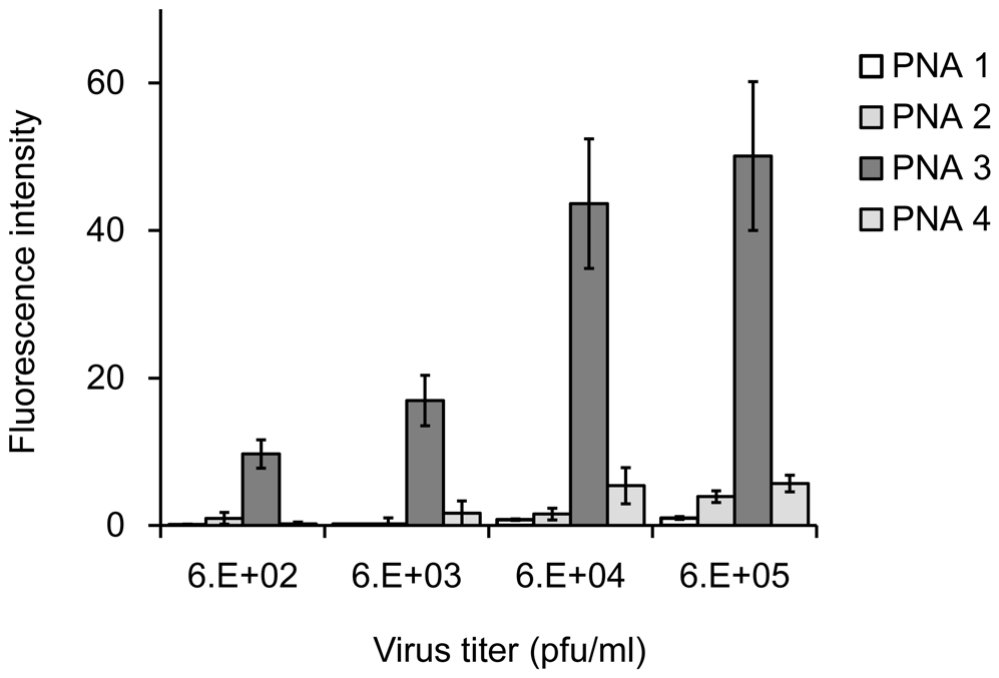
Fluorescence detection of the influenza A/Osaka/180/2009(H1N1) virus genome on PNA 1–4-immobilized plates. Conditions: 0.5 µg of PNA was immobilized on each well. Virus incubation: 100 µl of indicated virus solution, 1 h, r.t. Antibodies: 0.1 µg mouse monoclonal influenza A virus nucleoprotein primary antibody and goat anti-mouse IgG secondary antibody conjugated with horseradish peroxidase in each well, 2 h, r.t. Incubation of 3,3′,5,5′-tetramethyl-benzidene with horseradish peroxidase: 10 min, r.t. Fluorescence detection: excitation 430 nm, emission 503 nm.

PNA **1** did not capture the NS gene of A/H1N1pdm under these conditions (Fig. 3,) and PNA **2** captured only a small amount of the NS gene even at a virus concentration of 6 × 10^5^ pfu/ml (Fig. 3). In contrast, PNA **3** captured the NS gene much more effectively than PNA **2**, and the fluorescence signal increased in a virus concentration-dependent manner (Fig. 3). The fluorescence signal intensity observed in the PNA **3**-immobilized well was approximately 13-fold higher than that of PNA **2** at 6 × 10^5^ pfu/ml. PNA **4** was also immobilized on a plate and mismatch binding to the NS gene was monitored. Although a small increase in fluorescence signal was observed, it was much less than the signal intensity observed in the PNA **3**-immobilized well (Fig. 3). Clearly, the addition of a Hoogsteen strand to the N-terminus of PNA **1**, which possesses both a Watson-Crick strand and an AEEA linker, improved the binding efficiency to the target viral gene. Moreover, substituting the AEEA linker of bisPNA with an azobenzene amino acid dramatically enhanced binding affinity to the target gene without loss of sequence specificity.

### Sequence-specific Detection of Influenza A Virus Genome on a PNA-immobilized Plate

**Figure 4:**
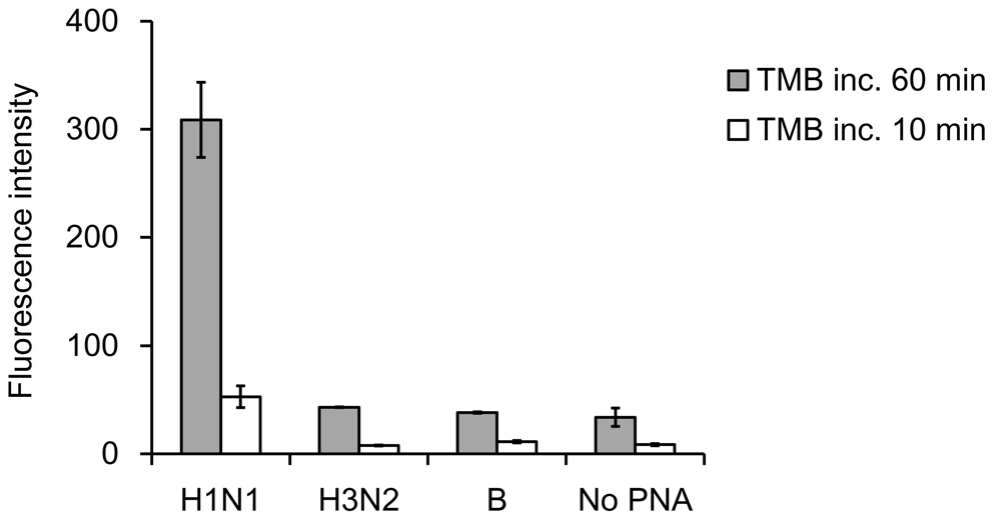
Detection of influenza A/Osaka/180/2009(H1N1), A/Panama/2007/99(H3N2), and B/Yamanashi/166/98 viruses on PNA 3-immobilized plate. Conditions: as for Fig. 3, except for the 3,3′,5,5′-tetramethyl-benzidene incubation times.

We further examined whether PNA **3** could discriminate the NS gene of the A/H1N1 virus from other virus subtypes. PNA **3**-immobilized wells were incubated with influenza A/Osaka/180/2009 (H1N1pdm), A/Panama/2007/99 (H3N2) or B/Yamanashi/166/98 virus. A/H1N1pdm was also incubated in PNA non-modified wells to evaluate non-specific binding of the NS gene to the well. We confirmed that PNA **3** selectively captured the NS gene of the influenza A/H1N1pdm virus (Fig. 4). There was no detectible crossover against the influenza A/H3N2 and B viruses; their fluorescence signal intensities were comparable to the background observed in the non-modified wells (Fig. 4, closed bar). The signal-to-noise ratio remained unchanged even upon longer incubation of TMB with HPR (Fig. 4), indicating that PNA **3** captured the NS gene of the influenza A/H1N1pdm virus in a sequence-specific manner.

### Visual Detection of Influenza A Virus Genome RNA on a PNA-immobilized Plate

**Figure 5:**
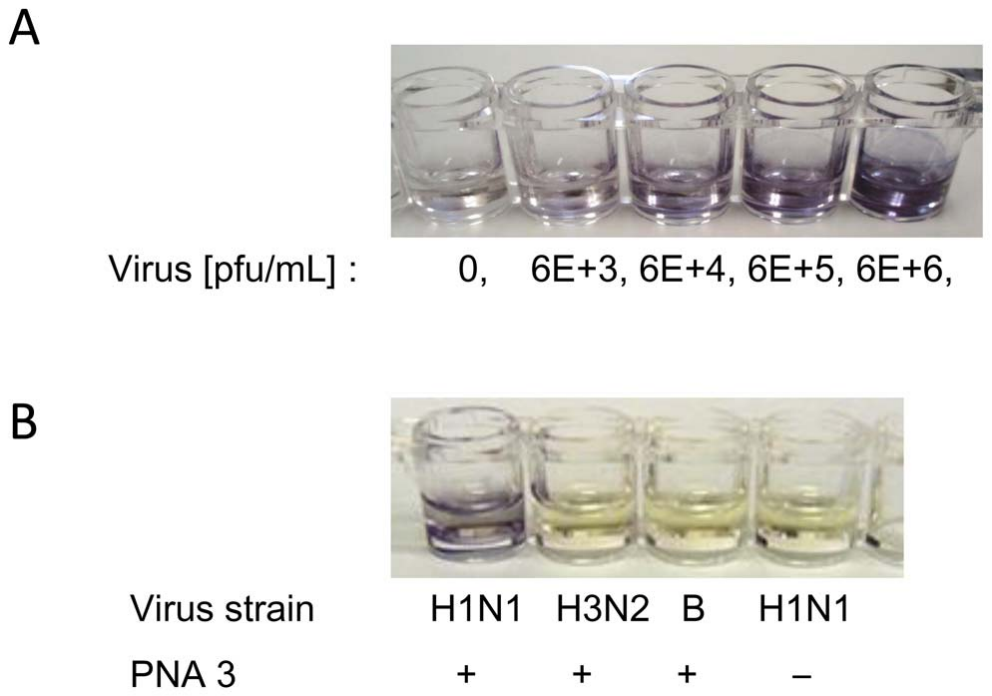
Visual detection of the NS gene of influenza A/Osaka/180/2009 (H1N1pdm) virus in wells containing immobilized PNA 3. A) Conditions: as for Fig. 3, but the virus concentration was 10-fold higher and the secondary antibody and the substrate were changed to anti-mouse IgG conjugated with alkaline phosphatase, and 5-bromo-4-chloro-3′-indolyphosphate and nitro-blue tetrazolium (BCIP/NBT), respectively. Incubation of BCIP/NBT with ALP: 60 min at r.t. B) Conditions: as for Fig. 4, except that the secondary antibody and the substrate were changed to anti-mouse IgG conjugated with alkaline phosphatase, and 5-bromo-4-chloro-3′-indolyphosphate and nitro-blue tetrazolium (BCIP/NBT), respectively. Incubation of BCIP/NBT with ALP: 60 min at r.t.

Our goal is to determine the presence of the NS gene of influenza A/H1N1pdm virus with the naked eye. We employed anti-mouse IgG secondary antibody conjugated with alkaline phosphatase in an ELISA assay using 5-bromo-4-chloro-3′-indolyphosphate and nitro-blue tetrazolium (BCIP/NBT) as substrates. Purple dye production increased in a virus concentration-dependent manner (Fig. 5A). The detection threshold was between 6.0 × 10^4^ and 6.0 × 10^5^ pfu/ml, the same range as the virus titer in clinical samples from influenza A virus-infected patients [15]. Purple dye production was evident upon incubation of the A/H1N1 virus in wells containing immobilized PNA **3** (Fig. 5B), while no dye production was observed when A/H3N2 and B viruses were incubated similarly, or when A/H1N1 virus was incubated in the control wells (Fig. 5B).

### Conclusions

The binding of oligonucleotides to influenza A virus genes is often hindered by the RNA tertiary structure and by bound proteins [16]. In this study we synthesized bisPNA-AZO, which is tethered via an AZO linker, and captured the influenza A virus NS gene without the need to purify the gene. The AZO linker improved the triplex formation efficiency of bisPNA by inducing Hoogsteen basepairing. BisPNA-AZO efficiently recognized the target virus gene sequence and inhibited its reverse transcription by MuLV reverse transcriptase. The inhibitory activity is approximately one order of magnitude higher than that of bisPNA-AEEA. Using a bisPNA-AZO-immobilized plate, we captured the target virus gene and visualized it by detecting the NP protein on the gene. The visual colour change is evident to the naked eye and is sensitive enough to allow the identification of influenza A virus in clinical samples. Since our detection system relies on the sequence-specific binding of bisPNA-AZO to the target virus gene, there was no cross-reactivity against other influenza virus subtypes. Although further optimization of the linker structure for bisPNA is required, this study demonstrates a fundamental approach for the direct visual identification of infectious virus pathogens by targeting their conserved gene sequences with a hairpin-type peptide nucleic acid. This method will be applicable to the identification of not only influenza A virus subtypes, but also drug-resistant strains caused by single-nucleotide polymorphisms (SNPs).

## Supporting Information

File S1 **Includes figures S1–S13.**
(DOC)
