# Supplementary material for: Sequence-Specific and Visual Identification of the Influenza Virus NS Gene by Azobenzene-Tethered Bis-Peptide Nucleic Acid
Source: PLoS One. 2013 May 21;8(5):e64017. doi: 10.1371/journal.pone.0064017 (PMC3660326; doi:10.1371/journal.pone.0064017)
Supplement: File S1 — Includes figures S1–S13. (DOC) [file pone.0064017.s001.doc]

Sequence-Specific and Visual Identification of the Influenza Virus NS Gene by Azobenzene-tethered bis-Peptide Nucleic Acid**

Kunihiro Kaihatsu*, Shinjiro Sawada, Shota Nakamura, Takaaki Nakaya, Teruo Yasunaga, Nobuo Kato

*Corresponding author.

^**^K.K and S.S contributed equally to this work.

**Supporting Information**

Table of Contents: S2

**Supplementary figure**

Figure S1 S3

**Supplementary materials and methods**

Chemicals S4

Preparation of Fmoc-Lys-(Boc)-OH loaded resin S4

PNA Solid phase synthesis S4

PNA purification and analysis S5

**Supplementary data**

HPLC analysis of PNA **1**-**6** (Fig. S2-S7) S7

MALDI-TOF-MS analysis of PNA **1**-**6** (Fig. S8-S13) S13

Abbreviations S16

***Figure S1.*** Effect of linker molecules for bisPNA on the melting profile when complexed with complementary DNA. PNA **2**-**4** were individually incubated with a complementary single strand DNA that contains the conserved sequence of the NS gene of influenza A/Osaka/180/63 (H1N1pdm). Conditions: 5 μM of PNA **2**, **3**, or **4** and 5 μM of DNA were dissolved in 10 mM phosphate buffer (pH 6.9). The temperature was ramped down from 95 to 10°C at a rate of -1°C/min.

**Supplementary materials and methods**

**Chemicals**

Fmoc/Bhoc-protected PNA monomers were purchased from Panagene (Daejeon, Korea). Fmoc-Lys(Boc)-OH, poly-ethylene linker and TGR-resin were purchased from Merck Millipore (Tokyo, Japan). The coupling activators, HBTU and HOBT, were purchased from Watanabe Chemicals (Hiroshima, Japan). PNA was immobilized via its 5’-terminal amino group to the surface of a 96 well enzyme-linked immunosorbent assay (ELISA) plate from Sumitomo Bakelite (Tokyo, Japan) by following the manufacture’s protocol. DNA (salt free) was purchased from Sigma-Genosys (Ishikari, Japan). Antibody for detecting influenza virus nucleoprotein was purchased from Lifespan Biosciences (Seattle, WA, USA). Peroxidase-labeled goat anti-mouse IgG and BCIP/NBT phosphate substrate were obtained from KPL (Gaithersburg, MS, USA). Peroxidase substrate (ECL plus solution containing 3,3',5,5'-tetramethyl-benzidenes (TMB)) was purchased from GE Healthcare (Tokyo, Japan). Alkaline phosphatase-conjugated goat polyclonal anti-mouse IgG was purchased from Thermo Fisher Scientific (Rockford, IL, USA). Other chemicals were purchased from Wako Pure Chemical (Osaka, Japan), Sigma-Aldrich (Tokyo, Japan) and Tokyo Chemical Industry (Tokyo, Japan). Reagents and solvents were used without further purification unless otherwise noted.

**Preparation of Fmoc-Lys-(Boc)-OH loaded resin**

The resin (200 mg, 34 μmol, 170 μmol/g) was swollen in 5 ml DMF for 30 min. To remove Fmoc, the resin was treated twice with 1.5 ml of DMF/piperidine (3:2, v/v) and then washed with DMF (5 × 3 ml). To preactivate, HBTU (12.9 mg, 34 μmol), HOBT (4.6 mg, 7.4 μmol), NMM (9.2 mg, 91 μmol) and pyridine (400 ml) were added to a mixture of Fmoc/Boc-protected lysine (Fmoc-Lys(Boc)-OH, 15.9 mg, 34 μmol) and Boc/Boc-protected lysine (Boc-Lys(Boc)-OH, 51.7 mg, 170 μmol) in DMF (1 ml). After 3 min, the mixture was added to the resin. After 60 min, the resin was washed with DMF (5 × 5 ml). For capping, the resin was treated twice with 1.5 ml of 2,6-lutidine/Ac2O/pyridine (6:5:89, v/v). The resin was washed with DMF (5 × 5 ml) and dried under reduced pressure.

**PNA solid phase synthesis**

Automated linear solid phase synthesis was performed using an Intavis ResPep parallel synthesizer equipped with micro scale columns (Köln, Germany) for PNA synthesis. To prepare the lysine-loaded resin, Novagen TGR Resin (200 mg, 34 μmol, 170 μmol/g) was swollen in 5 ml DMF for 30 min. To preactivate, HBTU (12.9 mg, 34 μmol), HOBT (4.6 mg, 7.4 μmol), NMM (9.2 mg, 91 μmol) and pyridine (400 ml) were added to a mixture of Fmoc/Boc-protected lysine (Fmoc-Lys(Boc)-OH, 15.9 mg, 34 μmol) and Boc/Boc-protected lysine (Boc-Lys(Boc)-OH, 51.7 mg, 170 μmol) in DMF (1 ml). After 3 min, the mixture was added to the resin. After 60 min, the resin was washed with DMF (5 × 5 ml). For capping, the resin was treated twice with 1.5 ml of 2,6-lutidine/Ac2O/pyridine (6:5:89, v/v), then washed with DMF (5 × 5 ml) and dried under reduced pressure. Fmoc-lysine loaded resin (50 mg, ca. 6.8 μmol) was swollen in DMF (2 ml). After 30 min, the resin was transferred to a synthesizer reactor and washed with DMF (5 × 200 μl). After extraction of DMF, Fmoc protecting groups were removed from the resin by 2, 2 min incubations in a solution of 40% (v/v) piperidine in DMF (200 μl), then the resin was washed with DMF (7 × 200 μl). For the coupling of monomers such as Fmoc-Bhoc-PNA-OH, Fmoc-AEEA-OH, Fmoc-AZO-OH and Fmoc-Lys(Boc)-OH, a preactivation vessel was charged with 30 mM monomer solution in DMF (510 μl), 90 mM HBTU and 90 mM HOBT solution in DMF (18 μl) and 90 mM NMM in pyridine (282 μl). After 2 min, 800 μl of pre-activation solution was transferred to the resin and incubated for 60 min. After extraction of the coupling solution, the resin was washed with DMF (5 × 200 μl). This coupling step was repeated twice. To cap the non-elongated amino groups, the resin was incubated with a solution (500 μl) consisting of Ac2O/2,6-lutidine/DMF (5:6:89, v/v/v) for 10 min and then washed with DMF (5 × 200 μl). Deprotection, coupling of the next monomer, and capping were repeated as described above until synthesis of the PNA molecule was completed. Prior to cleaving PNA molecules from the resin, the resin was washed with DMF (5 × 200 μl) followed by washing with isopropanol (5 × 200 μl). After drying the resin, a solution of TFA/m-cresol (4:1, v/v, 1 ml) was added and the mixture was incubated for 30 min. The resin was then filtered and washed with TFA/m-cresol (4:1, v/v, 1 × 200 μl). The eluents were combined in a tube and precipitated twice with ice-cold diethyl ether (2 ml). The precipitate was collected by centrifugation and the supernatant was discarded. The residue was dried under ambient atmosphere and then dissolved in distilled water (100 μl).

**PNA purification and analysis**

All PNAs were purified by reverse-phase HPLC using a JASCO PU-2086 pump system (Tokyo, Japan), a JASCO UV-2075 detector, a GL Science Inertsil 150 mm × 4.6 mm, 5 μm C-18 column for analytical runs, and a GL Science Inertsil, 250 mm × 20 mm, 3 μm C-18 column for semi-preparative runs. Eluting solvents (analytical: A (0.1% TFA in water) and B (0.1% TFA in acetonitrile); semi preparative: A (0.1% TFA in water) and B (0.1% TFA in acetonitrile)) were used in a linear gradient at a flow rate of 1 ml/min for analytical and 8 ml/min for semi preparative HPLC. Gradient for analytical runs: 0→50% B in 30 min (Fig. S9-S14). Gradient for semi-preparative runs: 0% B for 10 min, 0→5% B in 10 min, 5% B for 10 min, 5→10% B in 10 min, 10% B for 10 min, 10→20% B in 120 min, 20→50% B in 10 min. Detection was achieved with a UV-VIS-detector at 260 nm. PNA molecular weights were analyzed using an Ultraflex MALDI TOF/TOF Mass Spectrometer (Bruker Daltonics, Yokohama, Japan) (Fig. S15-S20). The optical density of PNA and DNA was measured at 260 nm with a UV1700 spectrometer (Shimadzu) using quartz cuvettes (4 × 10 mm). The extinction coefficient of PNA was calculated from the molar extinction coefficient obtained from http://www.panagene.com/. Measurements of absorption at 260 nm were carried out in a buffer solution (10 mM NaH2PO4, pH 7.0) at ambient temperature.

**Supplementary data**

**HPLC analysis of PNAs**

AEEA-CCTTCTCTTCCAGGA-Lys-(AEEA)_2_-Lys

| Peak No | Ret. Time | Area | Height | % Area |
| --- | --- | --- | --- | --- |
| 1 | 13.192 | 2364533 | 117161 | 99.083 |
| 2 | 14.333 | 10850 | 643 | 0.455 |
| 3 | 15.000 | 11033 | 613 | 0.462 |

Fig. S2. HPLC spectrum of PNA **1**.

(Lys)_3_-TCTCTTCC-AEEA-CCTTCTCTTCCAGGA-Lys-(AEEA)_2_-Lys


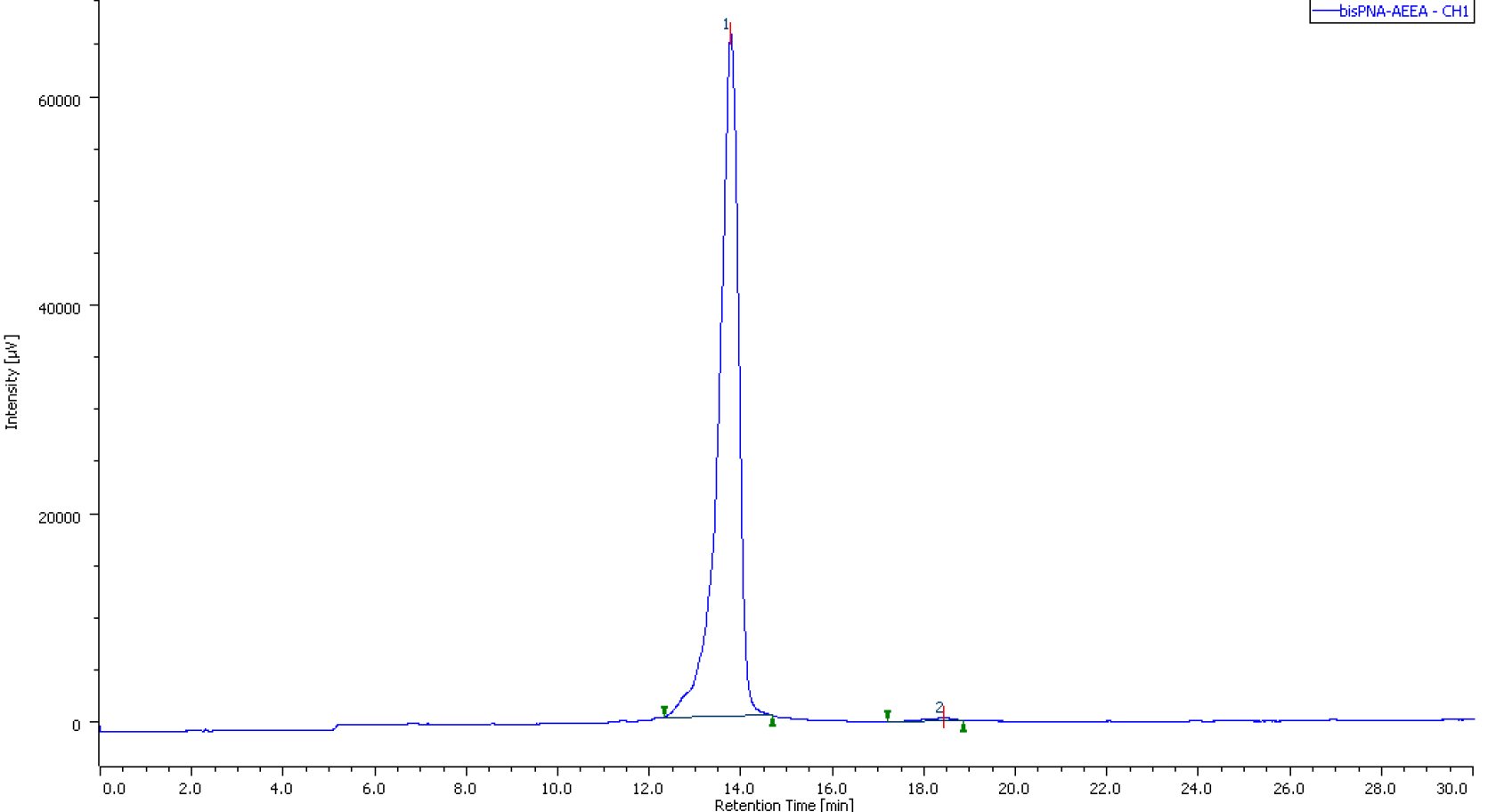


| Peak No | Ret. Time | Area | Height | % Area |
| --- | --- | --- | --- | --- |
| 1 | 13.767 | 1976225 | 65346 | 99.459 |
| 2 | 18.417 | 10759 | 238 | 0.541 |

Fig. S3. HPLC profile of PNA **2**.

(Lys)_3_-TCTCTTCC-AZO-CCTTCTCTTCCAGGA-Lys-(AEEA)_2_-Lys


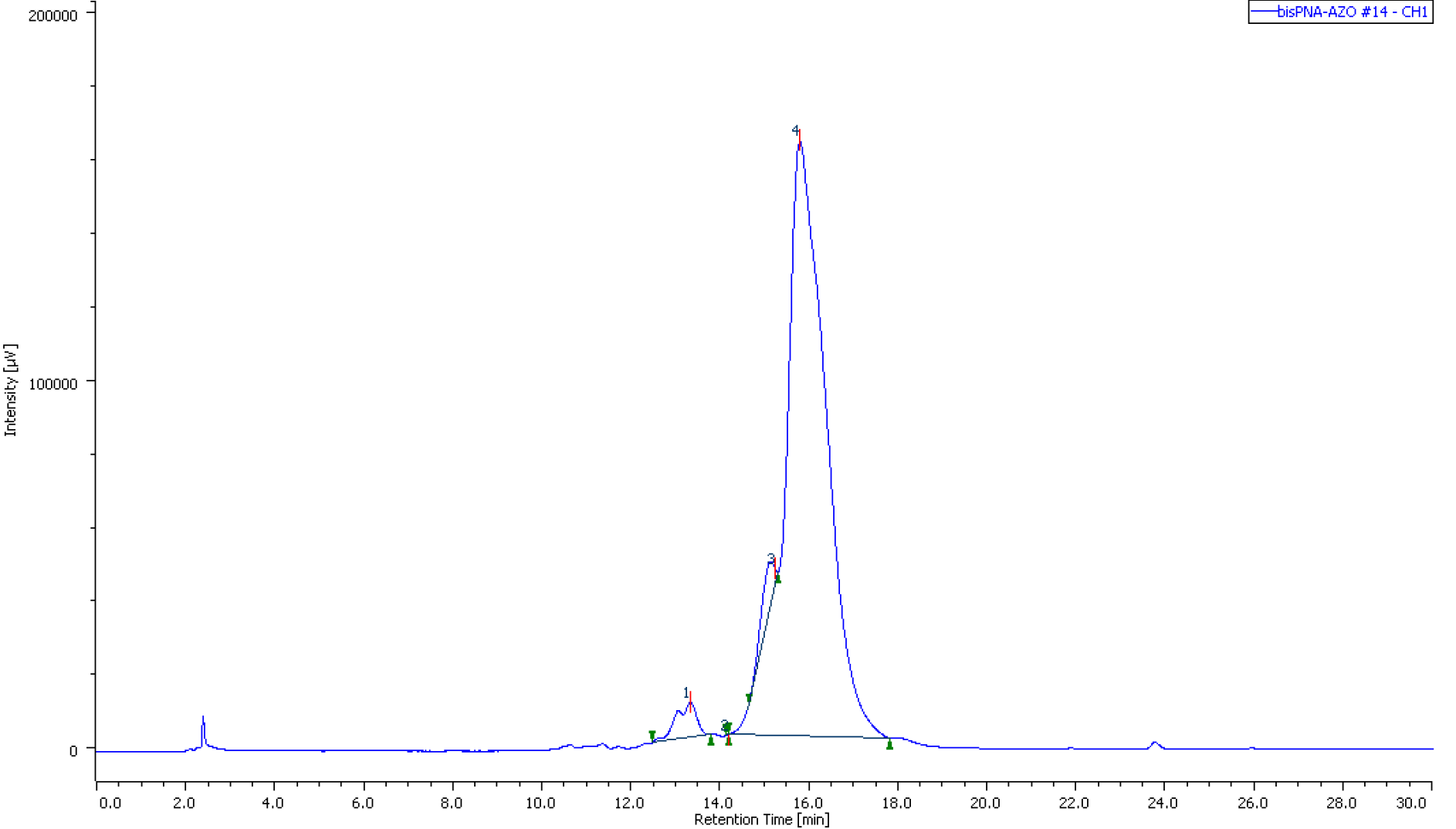


| Peak No | Ret. Time | Area | Height | % Area |
| --- | --- | --- | --- | --- |
| 1 | 13.333 | 302918 | 9367 | 2.735 |
| 2 | 14.175 | 73 | 13 | 0.001 |
| 3 | 15.225 | 259213 | 4634 | 2.341 |
| 4 | 15.783 | 10512771 | 161419 | 92.012 |

Fig. S4. HPLC profile of PNA **3**.

(Lys)_3_-TCTATTCC-AZO-CCTTATCTTCAAGGA-Lys-(AEEA)_2_-Lys


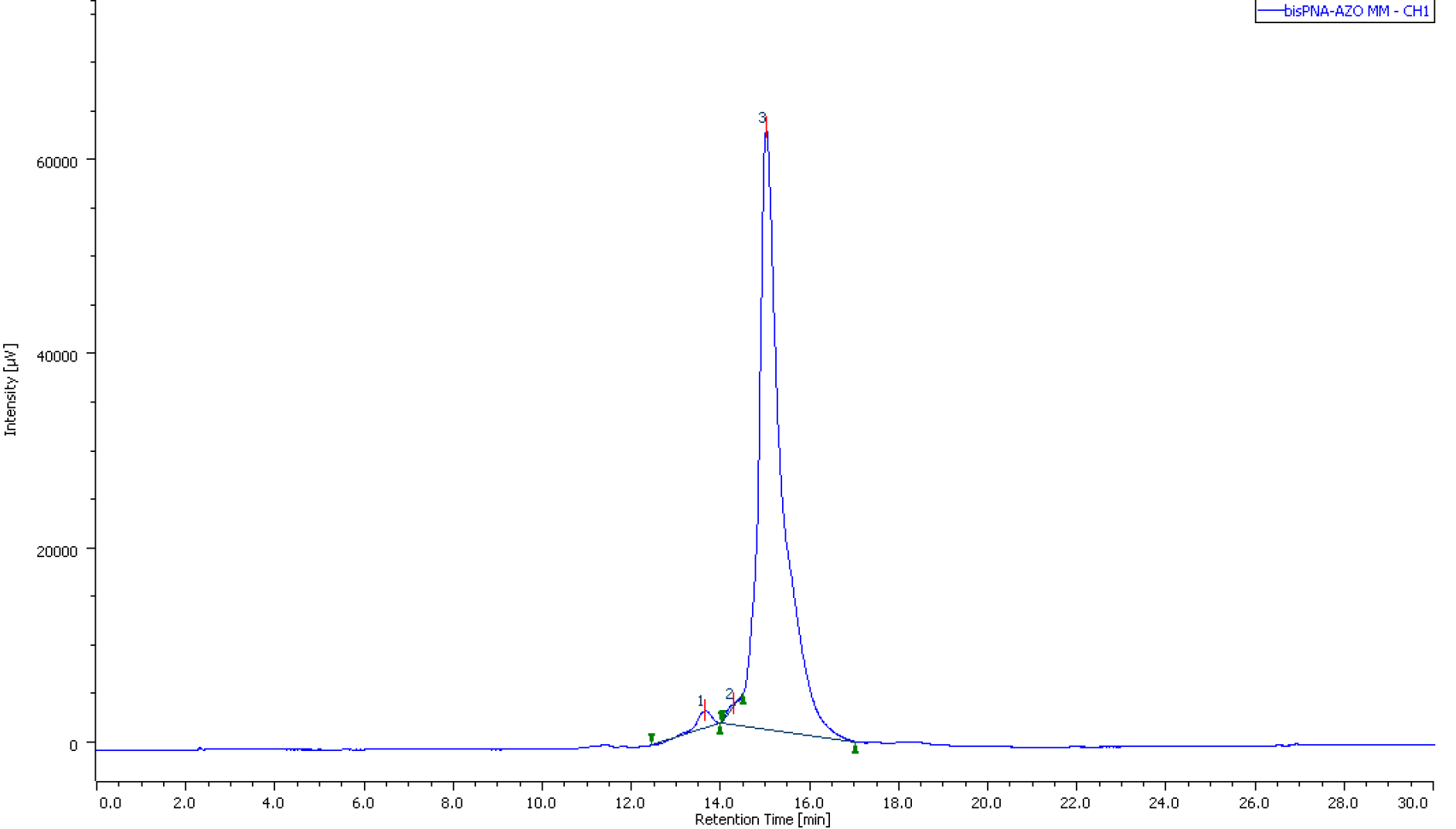


| Peak No | Ret. Time | Area | Height | % Area |
| --- | --- | --- | --- | --- |
| 1 | 13.642 | 41502 | 1762 | 1.801 |
| 2 | 14.275 | 5323 | 308 | 0.231 |
| 3 | 15.017 | 2257578 | 61837 | 97.968 |

Fig. S5. HPLC profile of PNA **4**.

(Lys)_3_-TTCCCTCC-AEEA-CCTCCCTTAGGTCAC-Lys-(AEEA)_2_-Lys

**
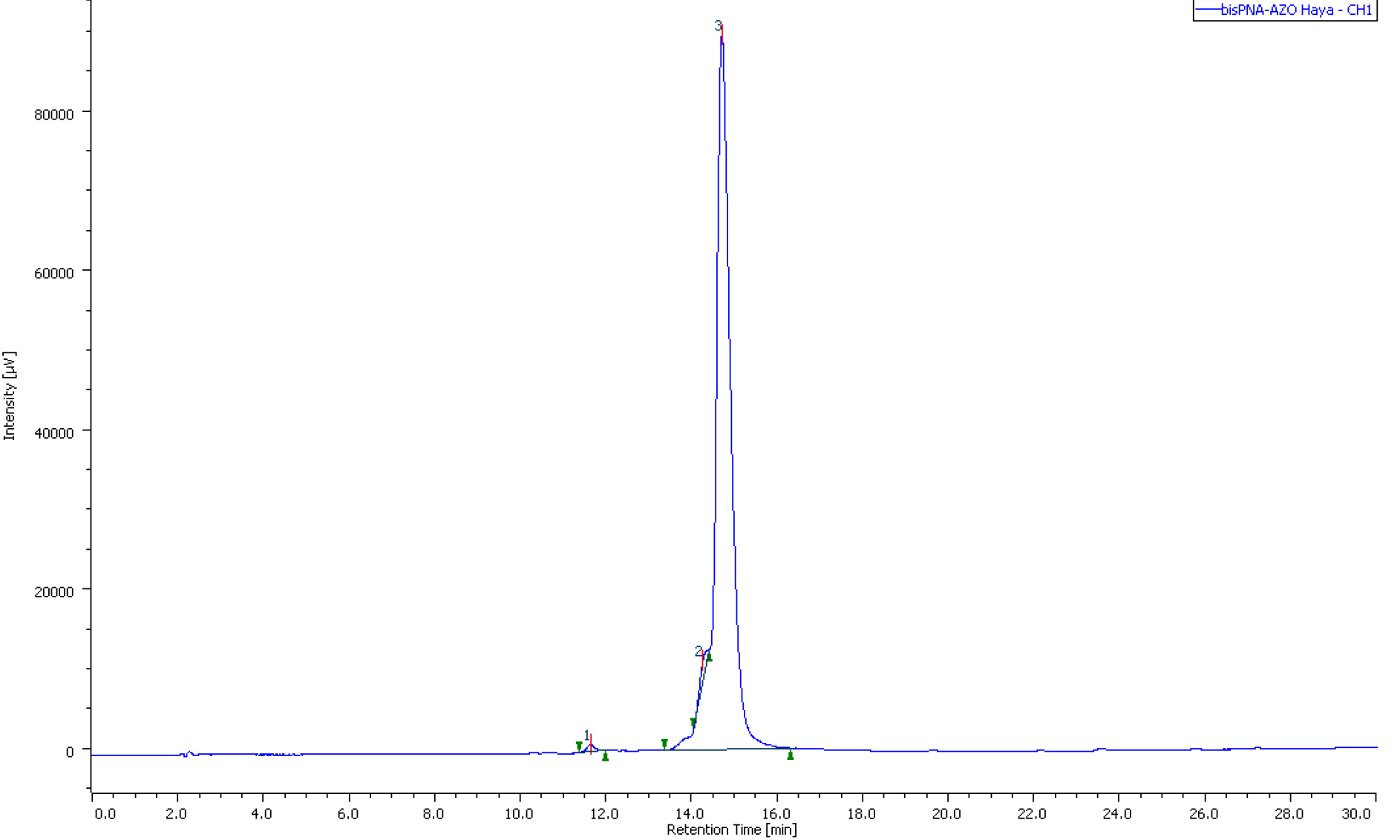
**

| Peak No | Ret. Time | Area | Height | % Area |
| --- | --- | --- | --- | --- |
| 1 | 11.558 | 44610 | 4332 | 1.737 |
| 2 | 12.000 | 31844 | 3761 | 1.240 |
| 3 | 12.333 | 12779 | 1537 | 0.498 |
| 4 | 12.992 | 248835 | 170458 | 96.525 |

Fig. S6. HPLC profile of PNA **5**.

(Lys)_3_-TTCCCTCC-AZO-CCTCCCTTAGGTCAC-Lys-(AEEA)_2_-Lys

**
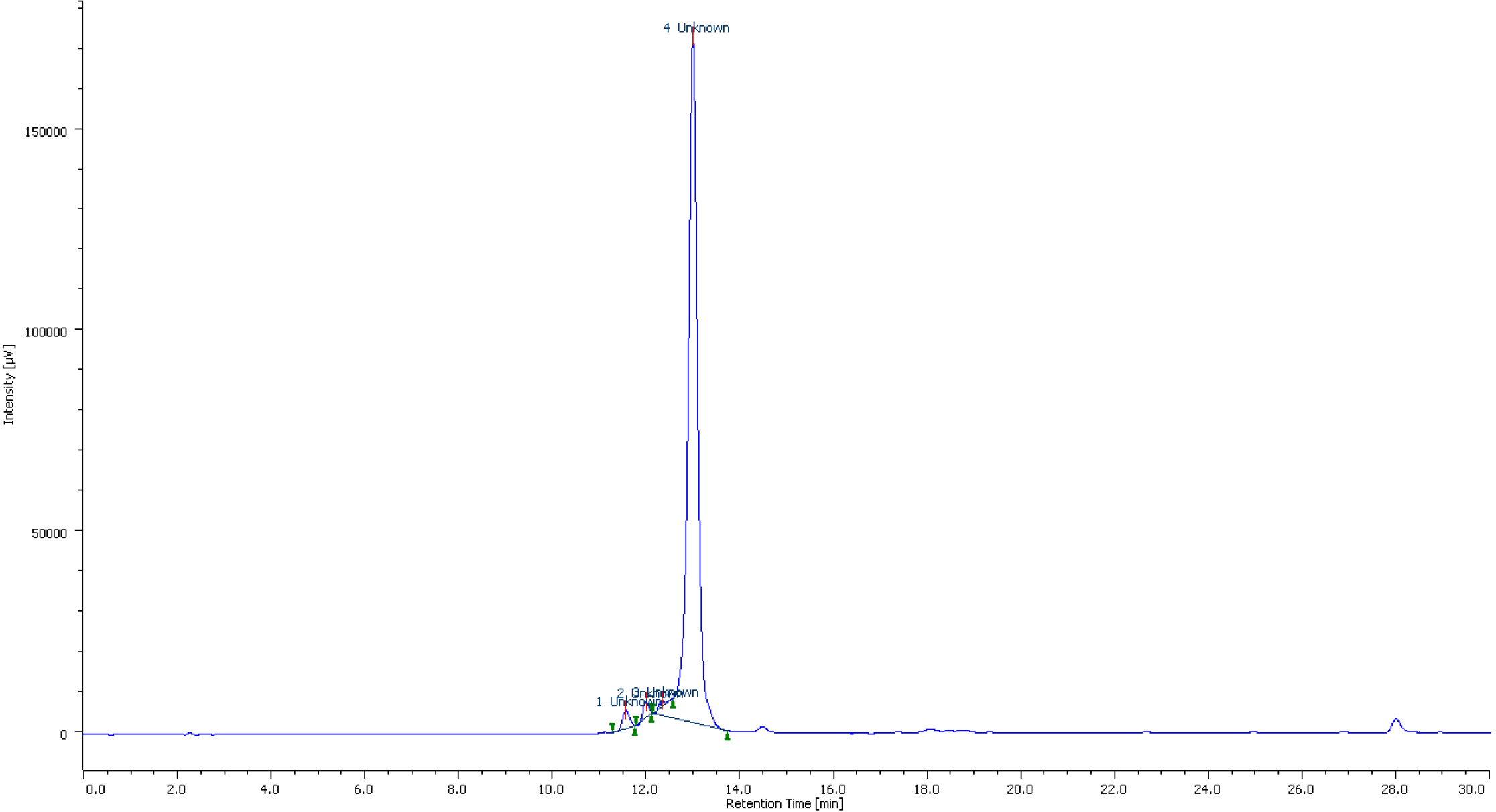
**

| Peak No | Ret. Time | Area | Height | % Area |
| --- | --- | --- | --- | --- |
| 1 | 11.633 | 12044 | 888 | 0.525 |
| 2 | 14.258 | 31338 | 2755 | 1.366 |
| 3 | 14.700 | 2250904 | 89606 | 98.109 |

Fig. S7. HPLC profile of PNA **6**.

**MALDI-TOF-MS analysis of PNAs**

AEEA-CCTTCTCTTCCAGGA-Lys-(AEEA)_2_-Lys (PNA **1**).

MALDI-TOF-MS (sinapinic acid): m/z calcd. 4682.14, found 4682.69 [M(average)]+.

Fig. S8. MALDI-TOF-MS profile of PNA **1**

(Lys)_3_-TCTCTTCC-AEEA-CCTTCTCTTCCAGGA-Lys-(AEEA)_2_-Lys (PNA **2**).

MALDI-TOF-MS (sinapinic acid): m/z calcd. 7136.88, found 7137.13 [M(average)]+.

Fig. S9. MALDI-TOF-MS profile of PNA **2**

(Lys)_3_-TCTCTTCC-AZO-CCTTCTCTTCCAGGA-Lys-(AEEA)_2_-Lys (PNA **3**).

MALDI-TOF-MS (sinapinic acid): m/z calcd. 7275.09, found 7299.05 [M(average)]+Na^+^.

Fig. S10. MALDI-TOF-MS profile of PNA **3**

(Lys)_3_-TCTATTCC-AZO-CCTTATCTTCAAGGA-Lys-(AEEA)_2_-Lys (PNA **4**).

MALDI-TOF-MS (sinapinic acid): m/z calcd. 7347.15, found 7378.55 [M(average)]+ +Na^+^.

Fig. S11. MALDI-TOF-MS profile of PNA **4**

(Lys)_3_-TTCCCTCC-AEEA-CCTCCCTTAGGTCAC-Lys-(AEEA)_2_-Lys (PNA **5**).

MALDI-TOF-MS (sinapinic acid): m/z calcd.7106.98, found 7106.27 [M(average)]+.

Fig.S12. MALDI-TOF-MS profile of PNA **5**

(Lys)_3_-TTCCCTCC-AZO-CCTCCCTTAGGTCAC-Lys-(AEEA)_2_-Lys (PNA **6**)

MALDI-TOF-MS (sinapinic acid): m/z calcd. 7245.19, found 7246.93 [M(average)]+.

Fig. S13. MALDI-TOF-MS profile of PNA **6**

**Abbreviations**

AEEA: 2-aminoethoxy-2-ethoxy acetic acid

BCIP: 5-bromo-4-chloro-3'-indolylphosphatase p-toluidine salt

Bhoc: benzhydryloxycarbonyl
Boc: tert-butyloxycarbonyl

cDNA: complementary DNA

DMF: N,N-dimethylformamide

Fmoc: 9-fluorenylmethoxycarbonyl

HBTU: 2-(1H-benzotriazole-1-yl)-1,1,3,3-tetramethyluronium hexafluorophosphate

HOBt: N-hydroxybenzotriazole

HPR: horseradish peroxidase

IgG: immunoglobulin G

NBT: nitro-blue tetrazolium chloride

NMM: N-methylmorpholine

NMP: N-methyl-2-pyrrolidon

TFA: trifluoroacetic acid

TMB: 3,3',5,5'-tetramethylbenzidine

Pfu: plaque formation unit

PNA: peptide nucleic acid

RNase: ribonuclease
